# Acute anterior uveitis and other extra-articular manifestations of spondyloarthritis

**Published:** 2015

**Authors:** TC Mitulescu, C Popescu, A Naie, D Predeţeanu, V Popescu, C Alexandrescu, LM Voinea

**Affiliations:** * Department of Internal Medicine and Rheumatology, “Carol Davila” University of Medicine and Pharmacy, Bucharest, Romania; ** ”Sfânta Maria” Clinical Hospital, Bucharest, Romania; *** University Emergency Hospital, Bucharest, Romania

**Keywords:** spondyloarthritis, uveitis, psoriasis, extra-articular manifestations

## Abstract

**Background:** Spondyloarthritis (SpA) is associated with an array of peripheral manifestations. Our study aims to evaluate extra-articular manifestations of SpA in a Romanian academic clinical setting and to observe their associations with different disease measures.

**Methods:** The study was designed to note the extra-articular manifestations of SpA patients in a cross-sectional and retrospective manner. Records included demographics, inflammation markers, SpA clinical characteristics, treatment regimes, associated osteoporosis and cardiovascular morbidity. Data were assessed by using appropriate non-parametric tests.

**Results:** A total of 126 SpA patients were included. The most common extra-articular manifestations were skin involvement in the form of psoriasis (34.1%), eye involvement in the form of acute anterior uveitis (8.7%) and dactylitis (7.2%). Compared to patients with no record of uveitis, uveitis-affected cases were more frequently males, more frequently diagnosed with ankylosing spondylitis, but less frequently dyslipidemic and diagnosed with psoriasis. Psoriasis-affected patients were older and had a higher prevalence of peripheral SpA diagnosis, but a lower prevalence of radiographic sacroiliitis.

**Conclusions:**Acute anterior uveitis in SpA predominantly affects males with AS. This is relevant both to clinical and fundamental science, since its management requires both ophthalmology and rheumatology clinical settings. Psoriasis was associated more frequently with peripheral SpA.

**Abbreviations:** AHT = arterial hypertension, AS = ankylosing spondylitis, ASAS = Assessment in SpondyloArthritis international Society, aSpA = axial spondyloarthritis, BASFI = Bath Ankylosing Spondylitis Functional Index, BASDAI = Bath Ankylosing Spondylitis Disease Activity Index, CRP = C-reactive protein, ESR = erythrocyte sedimentation rate, DM2 = type 2 diabetes mellitus, HLA = human leukocyte antigen, IBD = inflammatory bowel disease, MRI = magnetic resonance imaging, mSpA = mixed (peripheral and axial) spondyloarthritis, NSAIDs = non-steroidal anti-inflammatory drugs, pSpA = peripheral spondyloarthritis, PsA = psoriatic arthritis, ReA = reactive arthritis, SD = standard deviation, SI = sacroiliitis, SpA = spondyloarthritis, UDSpA = undifferentiated spondyloarthritis.

## Background

The spondyloarthritis (SpA) disease group encompasses several distinct nosological entities which have in common clinical, imagistic and genetic features: ankylosing spondylitis (AS), psoriatic arthritis (PsA), reactive arthritis (ReA), arthritis and/ or SpA associated with inflammatory bowel disease (IBD) and undifferentiated SpA (UDSpA). The initial recognition of the prototypical SpA, namely AS, was that of a inflammatory disease affecting the axial skeleton, as depicted by the modified New York diagnostic criteria [**1**]. Nevertheless, clinical evidence pointed out to an array of peripheral manifestations [**2**], which were soon included in classification criteria, first by Amor et. al. [**3**], then by the European Spondyloarthropathy Study Group [**4**], followed by the Assessment in SpondyloArthritis international Society (ASAS) [**5**,**6**]. The prevalence of these manifestations was amply documented by using data from national registries of SpA [**7**-**10**], which showed that extra-articular involvement in SpA has a broad range (for example, aside from plaque and palmoplantar psoriasis, skin involvement in SpA may also include erythema nodosum, pyoderma gangrenosum and keratoderma blennorrhagica) and should no be underestimated. Non-axial manifestations of SpA can be viewed as disease-specific (such as acute anterior uveitis, peripheral arthritis, dactylitis, enthesitis, psoriasis, urogenital disease and inflammatory bowel disease), reflecting the disease process at any time during its evolution, or as non-specific manifestations, reflecting the chronic inflammatory burden mostly during the later phases of the disease [**11**] and referring to bone (osteoporosis), pulmonary (upper lobe fibrosis, pleural thickening), cardiovascular (aoritis, aortic insufficiency, conduction abnormalities, elevated risk of acute coronary syndromes), renal (secondary amyloidosis, IgA nephropathy) and neurologic disease (cauda equina syndrome, impingement by spinal fracture or atlantoaxial subluxation) [**12**]. According to ASAS, non-axial manifestations of SpA should be monitored during different treatment regimes, since they can respond to therapy just as the axial manifestations [**13**] and since their resolution may indicate treatment efficacy and better quality of life [**14**,**15**]. In this context, our study aims to evaluate extra-articular manifestations of SpA in a Romanian academic clinical setting and to observe their associations with different disease measures.

## Materials and methods

**Study design**

The study was designed to observe the extra-articular manifestations of all the SpA patients admitted in the Department of Internal Medicine and Rheumatology of our hospital between January and June 2013 in a cross-sectional and retrospective manner, using medical records kept for each visit to the rheumatologist.

**Patients and data**

All the SpA patients included in the study met the following criteria: age above 18 years; Caucasian race; classification of SpA according to the physician’s diagnosis; no overlapping chronic inflammatory disease. The informed consent was presumed from the written informed consent each patient gave for being admitted to a university clinic (clinical examination, blood tests, imaging studies). The data were collected anonymously and the study was approved by the local ethics committee. Records included: demographics (age, gender, dwelling, smoking status); inflammation markers (C-reactive protein – CRP; erythrocyte sedimentation rate – ESR; fibrinogen – measured by commercial available kits); disease measures (type of SpA; disease duration; peripheral and non-articular involvement; human leukocyte antigen B27 status – HLA B27; disease activity estimated by using BASDAI [**16**] and functional impairment estimated by using BASFI [**17**]); treatment regimes (sulfasalazine, non-steroidal anti-inflammatory dugs – NSAIDs; anti-TNFα agents); associated osteoporosis (defined by a central T score < -2.5 or by specific antiresorptive drugs) and metabolic syndrome components (obesity defined by a body mass index ≥ 30 kg/ m2; dyslipidemia defined by abnormal values of cholesterol, its fractions and/ or triglycerides or by specific cholesterol lowering drugs [**18**]; arterial hypertension - AHT, defined by values ≥ 140/ ≥90 mmHg or by specific blood pressure lowering drugs [**19**]; type 2 diabetes mellitus – DM2, defined by fasting plasma glucose levels ≥ 126 mg/ dL, random plasma glucose ≥ 200 mg/ dL or by specific antidiabetic drugs) [**20**].

**Statistics**

Normally distributed data were reported as “mean (standard deviation)”, while non-normally distributed data were reported as “median (interquartile range)”. Differences were evaluated by using non-parametric tests: binomial and χ2 tests (or Fisher’s exact test where appropriate) for nominal data; Mann-Whitney U and Kruskal Wallis tests for scale data. Correlation was established by computing Spearman’s coefficients. All the tests were two-sided and considered significant if p ≤ 0.05 and were done by using SPSS Statistics v.17.0.1 for Windows (SPSS Inc., Chicago, U.S.A., 2008).

## Results

**General characteristics**

A total of 126 SpA patients met the inclusion criteria (**Table 1**).

**Table 1:** General characteristics of the group (n = 126)

*nominal variables*	*0*	*1*	*p*
sex (female/ male)	45 (35.7%)	81 (64.3%)	0.002
dwelling (rural/ urban)	38 (30.2%)	88 (69.8%)	< 0.001
smoking (no/ yes)	92 (73.1%)	34 (26.9%)	< 0.001
axial SpA (no/ yes)	9 (7.1%)	117 (92.9%)	< 0.001
peripheral SpA (no/ yes)	33 (26.2%)	93 (73.8%)	< 0.001
peripheral arthritis (no/ yes)	50 (39.7%)	76 (60.3%)	0.026
X-ray sacroiliitis (no/ yes)	78 (61.9%)	48 (38.1%)	0.010
MRI sacroiliitis (no/ yes)	120 (95.2%)	4 (3.2%)	< 0.001
HLA B27 (absent/ present)	7 (5.6%)	78 (61.9%)	< 0.001
NSAIDs (no/ yes)	52 (41.3%)	74 (58.7%)	0.061
sulfasalazine (no/ yes)	70 (55.6%)	56 (44.4%)	0.247
infliximab (no/ yes)	91 (72.2%)	35 (27.8%)	< 0.001
adalimumab (no/ yes)	106 (84.1%)	20 (15.9%)	< 0.001
etanercept (no/ yes)	104 (82.5%)	22 (17.5%)	< 0.001
AHT (no/ yes)	88 (69.8%)	38 (30.2%)	< 0.001
dyslipidemia (no/ yes)	83 (65.9%)	43 (34.1%)	< 0.001
DM2 (no/ yes)	116 (92.1%)	10 (7.9%)	< 0.001
obesity (no/ yes)	119 (94.4%)	7 (5.6%)	< 0.001
osteoporosis (no/ yes)	118 (93.6%)	8 (6.4%)	< 0.001
*scale variables*	*mean*	*interval*	*SD*
age (years)	39.7	21-61	11.1
disease duration (years)	9.1	0.25-37	8.4
ESR (mm/ h)	22.9	2-114	28.8
fibrinogen (mg/ dL)	309.7	172-577	112.5
CRP (mg/ L)	23.7	0-258	47.9
BASDAI	3.23	0-9.4	2.81
BASFI	3.32	0-22.5	3.72
*Note*: scale variables are reported as “mean (interval, SD)”, while nominal variables are reported as “frequency (subgroup percentage), p values represent the significance of the binomial test under the assumption of a 50% pre-test probability.			
*Abbreviations:* AHT = arterial hypertension, BASDAI = Bath Ankylosing Spondylitis Disease Activity Index, BASFI = Bath Ankylosing Spondylitis Functional Index, CRP = C-reactive protein, DM2 = type 2 diabetes mellitus, ESR = erythrocyte sedimentation rate, HLA = human leukocyte antigen, MRI = magnetic resonance imaging, NSAIDs = non-steroidal anti-inflammatory drugs, SpA = spondyloarthritis, SD = standard deviation.			

**Fig. 1:**
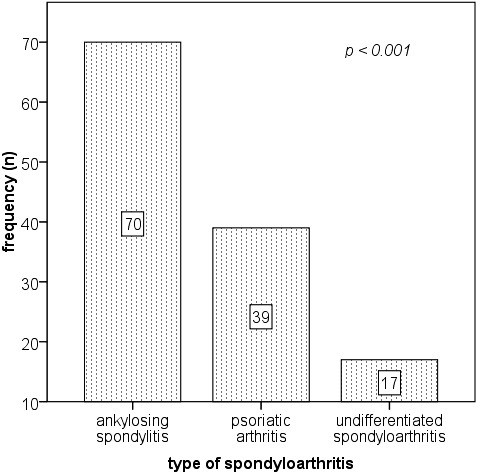
Spondyloarthritis phenotypes in the studies group. The p value represents the significance of the χ2 test

The dominant characteristics of the group were concisely represented by an active HLA B27 positive long-standing ankylosing spondylitis in men. Within the study period and clinical setting, there were no recoded cases of reactive arthritis and/ or spondylitis associated with IBD (**Fig. 1**). 

**Fig. 2:**
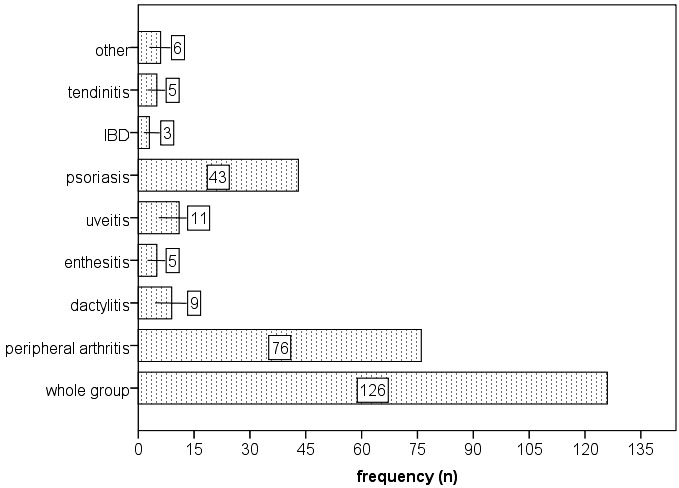
Types of extra-articular manifestations of spondyloarthritis in the studied group. The “other” category refers to chorea (1 case); plantar fasciitis (2 cases); nail dystrophy (3 cases). IBD – inflammatory bowel disease (Crohn disease, ulcerative colitis)

The most common extra-articular manifestations were skin involvement in the form of psoriasis (34.1%), eye involvement in the form of acute anterior uveitis (8.7%) and dactylitis (7.2%; **Fig. 2**). There were cases of neither amyloidosis, cardiovascular or pulmonary involvement, nor any cases of neurologic manifestations secondary to vertebral fracture/ fusion.

**Differences and associations of SpA-specific subgroups**

Compared PsA and UDSpA patients, AS patients had a higher prevalence of acute anterior uveitis and lower prevalence of psoriasis, IBD and tendinitis (**Table 2**). Also, patients with peripheral SpA tended to have a higher prevalence of psoriasis and tendinitis compared to patients with axial SpA. Regarding gender, males had acute anterior uveitis more frequently, while females had a higher prevalence of psoriasis. Interestingly, patients with radiographic sacroiliitis had a higher prevalence of enthesitis, but a lower prevalence of psoriasis compared to those without radiographic sacroiliitis.

**Table 2:** Significant differences between SpA subgroups

	AS (n=70)	PsA (n=39)	UDSpA (n=17)	p
uveitis (n)	10 (14.3%)	0 (0%)	1 (5.9%)	0.037
psoriasis (n)	3 (4.3%)	39 (100%)	1 (5.9%)	< 0.001
IBD (n)	1 (1.4%)	0 (0%)	2 (11.8%)	0.022
tendinitis (n)	0 (0%)	3 (7.7%)	2 (11.8%)	0.030
	aSpA (n=33)	pSpA (n=9)	mSpA (n=84)	
psoriasis (n)	4 (12.1%)	4 (44.4%)	35 (41.7%)	0.008
tendinitis (n)	1 (3%)	2 (22.2%)	2 (2.4%)	0.014
	female (n=45)	male (n=81)		
uveitis (n)	1 (2.2%)	10 (12.3%)		0.044
psoriasis (n)	21 (46.7%)	22 (27.2%)		0.027
	non-smoking (n=92)	smoking (n=34)		
psoriasis (n)	27 (29.3%)	16 (47.1%)		0.043
	non-aSpA (n=9)	aSpA (n=117)		
tendinitis (n)	2 (22.2%)	3 (2.6%)		0.041
	non-pSpA (n=33)	pSpA (n=93)		
psoriasis (n)	4 (12.1%)	39 (41.9%)		0.002
	no X-ray SI (n=78)	X-ray SI (n=48)		
psoriasis (n)	39 (50%)	4 (8.3%)		< 0.001
enthesitis (n)	1 (1.3%)	4 (8.3%)		0.049
	no arthritis (n=50)	arthritis (n=76)		
psoriasis (n)	4 (8%)	39 (51.3%)		< 0.001
enthesitis (n)	4 (8%)	1 (1.3%)		0.040
tendinitis (n)	0 (0%)	5 (6.6%)		0.044
other (n)	0 (0%)	6 (7.9%)		0.042
*Notes:* scale variables are reported as “median (intequartile range)”, while nominal variables are reported as “frequency (subgroup percentage)”; p values represent the significance of Mann Whitney (scale variables) or χ2 (nominal variables).				
*Abbreviations:* AS = ankylosing spondylitis, aSpA = axial spondyloarthritis, BASFI = Bath Ankylosing Spondylitis Functional Index, IBD = inflammatory bowel disease (Crohn disease, ulcerative colitis) mSpA = mixed (peripheral and axial) spondyloarthritis, PsA = psoriatic arthritis, pSpA = peripheral spondyloarthritis, SI = sacroiliitis, UDSpA = undifferentiated spondyloarthritis.				

**Differences and associations of extra-articular-specific subgroups**

Compared to patients with no record of uveitis, uveitis-affected cases were more frequently males, diagnosed with AS and treated with NSAIDs and sulfasalazine, but were less frequently dyslipidemic and less frequently diagnosed with psoriasis, PsA and UDSpA. Patients known with dactylitis, compared to those without dactylitis, had higher fibrinogen levels and higher rates of sulfasalazine treatment and tendinitis, but a lower rate of biologic treatment. Patients with psoriasis produced the most significant differences and associations (**Table 3**). 

**Table 3:** Significant associations of extra-articular manifestations

	no psoriasis (n=83)	psoriasis (n=43)	p
males (n)	59 (71.1%)	22 (51.2%)	0.027
smoking (n)	18 (21.7%)	16 (37.2%)	0.043
peripheral SpA (n)	54 (65.1%)	39 (90.7%)	0.002
peripheral arthritis (n)	37 (44.6%)	39 (90.7%)	< 0.001
X-ray sacroiliitis (n)	44 (53%)	4 (9.3%)	< 0.001
uveitis (n)	11 (13.3%)	0 (0%)	0.016
NSAIDs (n)	62 (74.7%)	12 (27.9%)	< 0.001
sulfasalazine (n)	46 (55.4%)	10 (23.3%)	0.001
obesity (n)	2 (2.4%)	5 (11.6%)	0.045
age (years)	38 (18)	50 (15)	< 0.001
*diagnosis*			
AS (n)	67 (80.7%)	3 (7%)	< 0.001
PsA (n)	0 (0%)	39 (90.7%)	
UDSpA (n)	16 (19.3%)	1 (2.3%)	
*type of SpA*			
axial SpA (n)	29 (34.9%)	4 (9.3%)	0.008
peripheral SpA (n)	5 (6%)	4 (9.3%)	
mixed SpA (n)	49 (59%)	35 (81.4%)	
	no uveitis (n=115)	uveitis (n=11)	
males (n)	71 (61.7%)	10 (90.9%)	0.044
psoriasis (n)	43 (37.4%)	0 (0%)	0.016
NSAIDs (n)	64 (55.7%)	10 (90.9%)	0.026
sulfasalazine (n)	47 (40.9%)	9 (81.8%)	0.011
dyslipidemia (n)	42 (36.5%)	1 (9.1%)	0.047
*diagnosis*			
AS (n)	60 (52.2%)	10 (90.9%)	0.037
PsA (n)	39 (33.9%)	0 (0%)	
UDSpA (n)	16 (13.9%)	1 (9.1%)	
	no dactylitis (n=117)	dactylitis (n=9)	
tendinitis (n)	3 (2.6%)	2 (22.2%)	0.041
sulfasalazine (n)	48 (41%)	8 (88.9%)	0.010
infliximab (n)	35 (29.9%)	0 (0%)	0.044
biologics (n)	74 (63.2%)	2 (22.2%)	0.029
fibrinogen (mg/ dL)	285 (139)	372 (203)	0.043
*Notes:*			
- scale variables are reported as “median (intequartile range)”, while nominal variables are reported as “frequency (subgroup percentage)”;			
- p values represent the significance of Mann Whitney (scale variables) or χ2 (nominal variables).			
*Abbreviations:* AS = ankylosing spondylitis, NSAIDs = non-steroidal anti-inflammatory drugs, PsA = psoriatic arthritis, SpA = spondyloarthritis, UDSpA = undifferentiated spondyloarthritis.			

Thus, compared to the no psoriasis subgroup, psoriasis-affected patients were older and had a higher prevalence of smoking, peripheral SpA diagnosis and obesity, but a lower prevalence of male gender, radiographic sacroiliitis, uveitis and treatment with NSAIDs and sulfasalazine. Compared to patients without tendinitis, those who were diagnosed with tendinitis had higher inflammation markers (ESR, fibrinogen), were more frequently diagnosed with peripheral SpA and obesity and had a higher prevalence of dactylitis, but had a lower disease duration and a lower rate of treatment with biologics (**Table 4**). 

**Table 4:** Significant associations of extra-articular manifestations

	no tendinitis (n=121)	tendinitis (n=5)	p
axial SpA (n)	114 (94.2%)	3 (60%)	0.041
peripheral SpA (n)	71 (58.7%)	5 (100%)	0.044
dactylitis (n)	7 (5.8%)	2 (40%)	0.041
biologics (n)	76 (62.8%)	0 (0%)	0.009
obesity (n)	5 (4.1%)	2 (40%)	0.025
disease duration (y)	7 (9)	1 (3)	0.016
ESR (mm/h)	14 (26)	49 (37)	0.005
fibrinogen (mg/ dL)	285 (134)	411 (199)	0.011
*diagnosis*			
AS (n)	70 (57.9%)	0 (0%)	0.030
PsA (n)	36 (29.8%)	3 (60%)	
UDSpA (n)	15 (12.4%)	2 (40%)	
*type of SpA*			
axial SpA (n)	32 (26.4%)	1 (20%)	0.014
peripheral SpA (n)	7 (5.8%)	2 (40%)	
mixed SpA (n)	82 (67.8%)	2 (40%)	
	no IBD (n=123)	IBD (n=3)	
HLA B27 (n)	78 (95.1%)	0 (0%)	< 0.001
obesity (n)	6 (4.9%)	1 (33.3%)	0.034
osteoporosis (n)	7 (5.7%)	1 (33.3%)	0.042
ESR (mm/ h)	15 (27)	43 (-)	0.015
*diagnosis*			
AS (n)	69 (56.1%)	1 (33.3%)	0.022
PsA (n)	39 (31.7%)	0 (0%)	
UDSpA (n)	15 (12.2%)	2 (66.7%)	
	no enthesitis (n=121)	enthesitis (n=5)	
peripheral arthritis (n)	75 (62%)	1 (20%)	0.040
X-ray sacroiliitis (n)	44 (36.4%)	4 (80%)	0.049
NSAIDs (n)	69 (57%)	5 (100%)	0.046
infliximab (n)	31 (25.6%)	4 (80%)	0.021
*Notes:*			
- scale variables are reported as “median (intequartile range)”, while nominal variables are reported as “frequency (subgroup percentage)”;			
- p values represent the significance of Mann Whitney (scale variables) or χ2 (nominal variables).			
*Abbreviations* AS = ankylosing spondylitis, ESR = erythrocyte sedimentation rate, HLA = human leukocyte antigen, IBD = inflammatory bowel disease (Crohn disease, ulcerative colitis), NSAIDs = non-steroidal anti-inflammatory drugs, PsA = psoriatic arthritis, SpA = spondyloarthritis, UDSpA = undifferentiated spondyloarthritis.			

Patients with IBD, compared to those without IBD, had higher inflammation markers and a higher prevalence of obesity, osteoporosis and UDSpA, but lower prevalence of HLA B27, AS and PsA. Patients diagnosed with enthesitis, compared to those without enthesitis, had a higher prevalence of radiographic sacroiliitis, NSAIDs and infliximab treatment, but a lower prevalence of peripheral arthritis. 

## Discussion

**Main findings**

As our data showed, acute anterior uveitis in SpA is predominantly a disease of male AS patients [**21**]. The fundamental science question regarding the association of acute anterior uveitis and AS seems to be answered by their independent associations with HLA B27 [**22**]. Such an explanation lacks in the case of male gender - acute anterior uveitis association within the SpA subpopulation, knowing that, when ignoring the cause of acute anterior uveitis, females seem to have a higher prevalence of uveitis in the general population [**23**]. One explanation would be that simply SpA is more frequent among males, but this would only explain incidence, not prevalence of acute anterior uveitis. Since male gender is associated with higher prevalence and severity of uveitis in other immune-mediated chronic diseases, such as Behçet’s disease [**24**] and juvenile idiopathic arthritis [**25**], we may hypothesize that the observation is explained by the different gender-specific hormone pattern which influences immune cell activity and mediator production (cytokines, immunoglobulins). For example, it is known that granulocyte chemotaxis is decreased by estrogen and is not influenced by testosterone [**26**]. The fact that our uveitis-diagnosed SpA patients had a lower prevalence of dyslipidemia is not conveniently explained by age, as one would have expected. In fact in our retrospective study, patients with uveitis were non-significantly older than the non-uveitis patients (median age of 42 years compared to 38 years; p = 0.176). The determinism in this case was bidirectional and insolvable in the study setting: either chronic inflammation led independently to both uveitis and dyslipidemia (which was more plausible), or there was a cryptic pathogenic link between lipid metabolism and susceptibility of uveitis (which was not implausible since phospholipids exhibited a crucial role in cell membrane function [**27**]). The observation that uveitis-patients were less frequently diagnosed with psoriasis should not deny the link between the two immune-mediated pathological conditions. Our data simply contained a higher proportion of AS patients in the non-uveitis subgroup, which was enough to explain the difference.

Recently, Thom et al. [**28**] reported that, compared to non-psoriasis subjects, those with psoriasis had a significantly higher prevalence of SpA, but also a significantly higher prevalence of axial pain and inflammatory back pain. Interestingly, our data showed quite the opposite, that psoriasis was more frequently associated with peripheral SpA and was less frequently associated with radiographic sacroiliitis. This difference is explained by the fact that the cited authors studied a normal sample, whereas we have focused on SpA-diagnosed patients. Still, the question remains: is psoriasis a marker of peripheral SpA? A controlled prevalence study would be appropriate to assess this further research question.

**Study limitations**

The study had several limitations, which may raise some difficulties in interpreting the results. First, the retrospective design had to deal with missing and real-life data and with the lack of a thoroughly controlled-study design. The period was short, since there were no cases of reactive and IBD-related arthritis. The nosological entities recorded by the study were solely classified as such according to the physician’s opinion. There were no normal subjects to compare the prevalence of different independent conditions such as uveitis and psoriasis.

## Conclusions

Acute anterior uveitis in SpA predominantly affects males with AS. This is relevant both to clinical and fundamental science. In ophthalmology clinical settings, a case of acute anterior uveitis in a young male patient should prompt the clinician to take into account a possible SpA differential diagnosis, especially in the presence of chronic back pain. Likewise, in rheumatology clinical settings, a case of acute anterior uveitis in an AS patient should indicate to the physician either a disease flare and/ or treatment inadequacy. In our study group, psoriasis was associated more frequently with peripheral SpA.

**Conflicts of interest**

None declared.

**Acknowledgements**

This paper was partly supported by the Sectorial Operational Programme Human Resources Development (SOPHRD), financed by the European Social Fund and the Romanian Government under the contract number POSDRU 141531. The authors would like the thank physicians from the “Sfânta Maria” Clinical Hospital, Bucharest, Clinic of Internal Medicine and Rheumatology, for providing access to the patients’ records.
